# Clinical impact of genomic characterization of 15 patients with acute megakaryoblastic leukemia–related malignancies

**DOI:** 10.1101/mcs.a005975

**Published:** 2021-04

**Authors:** Emilie Lalonde, Stefan Rentas, Gerald Wertheim, Kajia Cao, Lea F. Surrey, Fumin Lin, Xiaonan Zhao, Amrom Obstfeld, Richard Aplenc, Minjie Luo, Marilyn M. Li

**Affiliations:** 1 Department of Pathology and Laboratory Medicine, Children's Hospital of Philadelphia, Philadelphia, Pennsylvania 19104, USA;; 2 Department of Pathology and Laboratory Medicine, Perelman School of Medicine, University of Pennsylvania, Philadelphia, Pennsylvania 19104, USA;; 3 Department of Pediatrics, Perelman School of Medicine, University of Pennsylvania, Philadelphia, Pennsylvania 19104, USA

**Keywords:** acute megakaryocytic leukemia

## Abstract

Acute megakaryoblastic leukemia (AMKL) is a rare subtype of acute myeloid leukemia but is approximately 500 times more likely to develop in children with Down syndrome (DS) through transformation of transient abnormal myelopoiesis (TAM). This study investigates the clinical significance of genomic heterogeneity of AMKL in children with and without DS and in children with TAM. Genomic evaluation of nine patients with DS-related TAM or AMKL, and six patients with non-DS AMKL, included conventional cytogenetics and a comprehensive next-generation sequencing panel for single-nucleotide variants/indels and copy-number variants in 118 genes and fusions involving 110 genes. Recurrent gene fusions were found in all patients with non-DS, including two individuals with complex genomes and either a *NUP98–KDM5A* or a *KMT2A*–*MLLT6* fusion, and the remaining harbored a *CBFA2T3–GLIS2* fusion, which arose from both typical and atypical cytogenetic mechanisms. These fusions guided treatment protocols and resulted in a change in diagnosis in two patients. The nine patients with DS had constitutional trisomy 21 and somatic *GATA1* mutations, and those with DS-AMKL had two to four additional clinically significant somatic mutations. Comprehensive genomic characterization provides critical information for diagnosis, risk stratification, and treatment decisions for patients with AMKL. Continued genetic and clinical characterization of these rare cancers will aid in improving patient management.

## INTRODUCTION

Acute megakaryoblastic leukemia (AMKL) is a rare subtype of acute myeloid leukemia (AML), defined by the presence of at least 50% of blasts from the megakaryocytic lineage, and patients often present with thrombocytopenia or thrombocytosis. Other clinical features include dysplastic neutrophils, erythroid cells, and germ cell tumors in young men. AMKL is typically observed in children, accounting for 4%–15% of AML cases, compared to <1% of adult patients. A diagnosis of AMKL can be made if the pathognomonic t(1;22)(p13.3;q13.3) translocation is observed, resulting in a *RBM15–MKL1* gene fusion, or by pathological assessment of bone marrow as described above. However, recent studies have shown that AMKL is genetically heterogeneous with distinct subgroups based on cytogenetic and molecular alterations (Hama et al. 2008; Bhatnagar et al. 2016).

Children with Down syndrome (DS) have an estimated 150- to 500-fold increased risk of AMKL compared to children without DS (Creutzig et al. 1996; Hasle et al. 2000). Somatic *GATA1* mutations drive the development of transient abnormal myelopoiesis (TAM; also known as transient myeloproliferative disorder [TMD]) in utero through impaired megakaryocytic differentiation (Roy et al. 2009; Bhatnagar et al. 2016). Although most patients undergo spontaneous resolution of TAM within weeks of diagnosis, a small subset of high-risk patients presenting with life-threatening signs such as respiratory impairment, hepatic dysfunction, and/or leukocytosis (white blood cell [WBC] > 100,000) may not survive (Gamis et al. 2011). A further 20% of patients with TAM will go on to develop DS-AMKL due to accumulation of additional genetic mutations and clonal expansion (Nikolaev et al. 2013; Yoshida et al. 2013).

Genetic analysis of non-DS AMKL has identified additional recurrent gene fusions in addition to the pathognomonic *RBM15–MKL1* fusion. Next-generation sequencing (NGS) and targeted analysis of genes commonly altered in myeloid malignancies revealed a number of recurrent genetic rearrangements that evaded cytogenetic detection (Gruber et al. 2012; de Rooij et al. 2017). Evaluation of 89 pediatric patients with non-DS AMKL using NGS revealed an inversion on Chromosome 16 associated with a *CBFA2T3–GLIS2* gene fusion in 18% of patients, rearrangements involving *KMT2A* in 17% of patients, rearrangements of the *HOX* gene cluster in 14% patients, and a t(11;12) translocation resulting in a *NUP98–KDM5A* gene fusion in 11.5% of patients (De Rooij et al. 2017). Interestingly, the pathognomonic t(1;22)(p13.1;q13.3) translocation is observed in only 10% of patients, and acquired GA*TA1* mutations, which arise in all TAM patients, are observed in 9% of patients of non-DS AMKL. In addition to the stark differences in genomic profiles between DS-AMKL and non-DS AMKL, the latter is also associated with significantly worse prognosis and higher mortality rates (Hama et al. 2008; de Rooij et al. 2016).

The recurrent genetic features carry different diagnostic, prognostic, and therapeutic implications, making comprehensive genetic testing critical for optimal patient management. We present 15 patients with AMKL-related malignancies that illustrate various clinical scenarios and genomic strategies enabling accurate diagnosis and prognostication of AMKL.

## RESULTS

### AMKL Not Related to Down Syndrome (Non-DS AMKL)

Six patients were diagnosed with AMKL unrelated to DS, of which five presented between 1 to 2 yr of age, with physical symptoms including fevers, bruising, rashes, and cytopenia (Table 1). The sixth patient presented at 11 yr of age with persistent thrombocytopenia during routine follow-up 5 yr post low-risk B-cell acute lymphoblastic leukemia (B-ALL) treatment. Given that this patient's prior therapy for B-ALL did not include any cytotoxic therapies known to be associated with therapy-related AML, it was hypothesized that his AMKL may be a de novo AML, although the possibility of a therapy-related AML could not be excluded. Clinical history for all patients is summarized in Table 1. Pathological evaluation was suggestive of AMKL in four of six cases, but genetic testing was required for diagnosis in patients 1 and 3 (see Discussion).

**Table 1.** Clinical characteristics

Patient	Group	Age at diagnosis	Sex	Pathological diagnosis	Flow cytometry positive markers (of selected set^a^)	Clinical indication for genetic testing	Clinical presentation	Treatment	Follow-up status	Time to event
1	Non-DS AMKL	32 mo	M	AML, not otherwise specified	All negative	Leukemia	Unexplained bruising, leg discomfort, and some night-time sweating; marked anemia and thrombocytopenia with concern for malignancy	Matched unrelated BMT per AAML0531 arm B	Relapsed	9 mo
2	Non-DS AMKL	23 mo	F	Acute myeloid (megakaryoblastic) leukemia	CD41^+^, CD56^+^, CD61^+^	AML	6 wk of fevers, viral illness, ear infections	Matched unrelated BMT per AAML0531 arm B; MRD positive (0.8%) at the time of BMT	Deceased	8.5 mo
3	Non-DS AMKL	17 mo	M	Myeloid sarcoma (cranial mass), AML (bone marrow)	CD41^+^, CD56^+^, CD61^+^	Cranial mass	Soft tissue/bony mass of the left temporal area with intracranial extension and mass effect later diagnosed as a myeloid sarcoma	Matched-unrelated BMT per AAML0531 arm B; proton radiation to sarcoma	Remission	9 mo
4	Non-DS AMKL	18 mo	M	AMKL	CD41^+^, CD56^+^, CD61^+^	AML/ megakaryo-blastic leukemia, abnormal CBC	Extensive petechial rash on hands spread to legs/feet, easy bruising, low platelets, and pancytopenia	NA—treated at outside hospital	NA	NA
5	Non-DS AMKL	23 mo	F	Relapsed/recurrent AMKL	CD41^+^, CD56^+^, CD61^+^	Relapsed/recurrent AML	Early medullar relapse of AML, day 15 post reinduction	Matched unrelated BMT; conditioned with busulfan, fludarabine, and anti-thymocyte globulin	Remission	2 mo
6	Non-DS AMKL	11 yr	M	AMKL	CD41^+^, CD42b dim, CD61^+^	History of low-risk ALL, now with concern for relapsed leukemia	Progressive thrombocytopenia concerning for relapsed B-ALL (70 mo from treatment of low-risk B-ALL)	Planned BMT; AAML0531 arm B-like	Undergoing treatment	NA
7	TAM	2 wk	M	NA	CD41^+^, CD56^+^, CD61^+^, CD7^+^	TAM plus presumed trisomy 21; 47, XY, +21.	Concern for germline GATA1	NA—treated at outside hospital	NA	NA
8	TAM	6 wk	M	NA	CD41subset, CD42b subset, CD61subset, CD7 subset	6 wk old with trisomy 21, TAM	Preterm with congenital heart defect, ascites, hepatic dysfunction, disseminated intravascular coagulation, and leukocytosis	Low-dose cytarabine	Deceased from cardiac and ascites complications	2 mo
9	TAM	2 d	M	NA	CD41subset, CD7^+^, CD56 subset, CD61subset	Concern for acute leukemia, TAM	Cardiorespiratory failure, leukocytosis with >70% circulating blasts, with Hgb ∼ 10 and platelets ∼ 200	Low-dose cytarabine	Remission	20 mo
10	TAM	20 d	M	NA	CD41subset, CD42b subset, CD56^+^, CD61subset, CD7^+^	TAM and presumed trisomy 21	Concern for TAM and respiratory distress; WBC 57,600	None	Remission	12 mo
11	DS-AMKL	2 yr	M	NA	CD41^+^, CD61^+^, CD7^+^	Myeloid leukemia of DS	Persistent thrombocytopenia	COG AAML1531	Remission	9 mo
12	DS-AMKL	14 mo	M	Myeloid leukemia associated with DS	CD41variable, CD56 subset, CD61variable, CD7^+^	New diagnosis AMKL with history of TAM	Patient with trisomy 21 who previously had TAM (not requiring chemoreduction)	COG AAML1531	Remission	8.5 mo
13	DS-AMKL	25 mo	M	Myeloid leukemia associated with DS	CD38^+^, CD41^+^, CD56 subset, CD61^+^, CD7^+^, MPO^+^	25 mo old with trisomy 21 and new AMKL	Trisomy 21 with hyperleukocytosis, profound anemia, thrombocytopenia, and splenomegaly	COG AAML1531	Remission	26.5 mo
14	DS-AMKL	18 mo	M	Myeloid leukemia associated with DS	CD41subset, CD56^+^, CD61subset, CD7^+^	New diagnosis leukemia	Irritability, significant jaundice, splenomegaly, and petechial rash; WBC 21.2, hgb 4.0, platelets 32, and 52% circulating blasts	COG AAML1531	Remission	26 mo
15	DS-AMKL	15 mo	F	Myeloid leukemia associated with DS	CD41^+^, CD42b^+^, CD61^+^, CD7^+^	Myeloid leukemia associated with DS	Irritability, poor feeding, and recurring fevers	COG AAML1531 intensification II (MRD 2.4% at the end of induction I; switched to Arm B)	Remission	20.5 mo

Blast counts, when present, refer to total blasts seen.

(AMKL) Acute megakaryoblastic leukemia, (AML) acute myeloid leukemia, (B-ALL) B-cell acute lymphoblastic leukemia, (BMT) bone marrow transplant, (COG) Children's Oncology Group, (d) days old, (DS) Down syndrome, (mo) months old, (MRD) minimal residual disease, (TAM) transient abnormal myelopoiesis, (y) years old.

^a^Selected markers include CD2, CD38, CD41, CD42b, CD56, CD61, CD7, MPO, and TDT. Only positive markers are listed; the remaining are negative, except in patient 8 for whom markers TdT, CD2, CD56, and CD38 were not evaluated.

**Figure 1.**
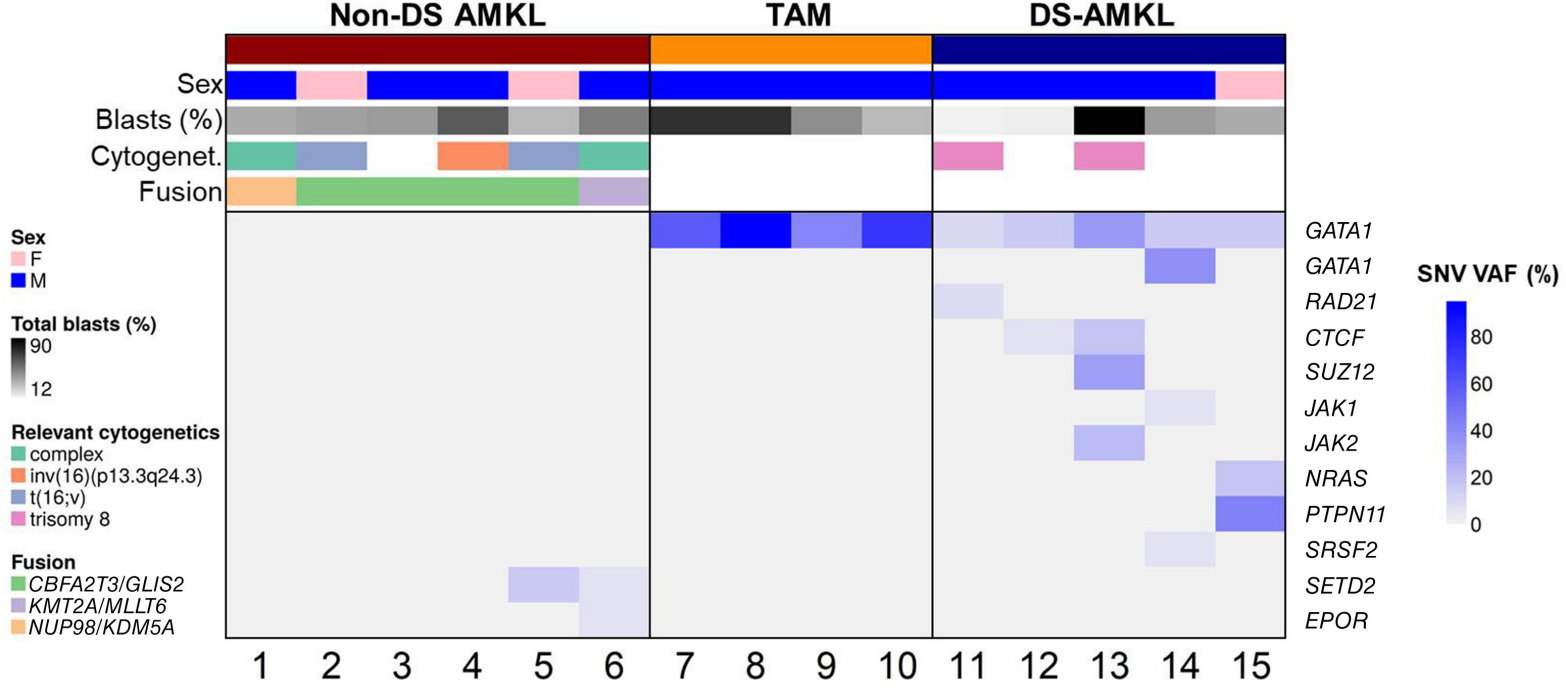
Genomic profile of 15 patients with acute megakaryocytic leukemia (AMKL)-related malignancies. Patients with non-Down syndrome AMKL (non-DS AMKL) are defined by recurrent gene fusions, whereas patients with transient myeloproliferative disease (TAM) have *GATA1* mutations with high variant allele fraction (VAF), and patients with DS-AMKL have additional somatic variants in signaling pathway genes. Total blast percentages are based on flow cytometry. Only variants classified as Tier 1–2 are listed. See Table 2 for complete genomic profile of each individual.

**Figure 2.**
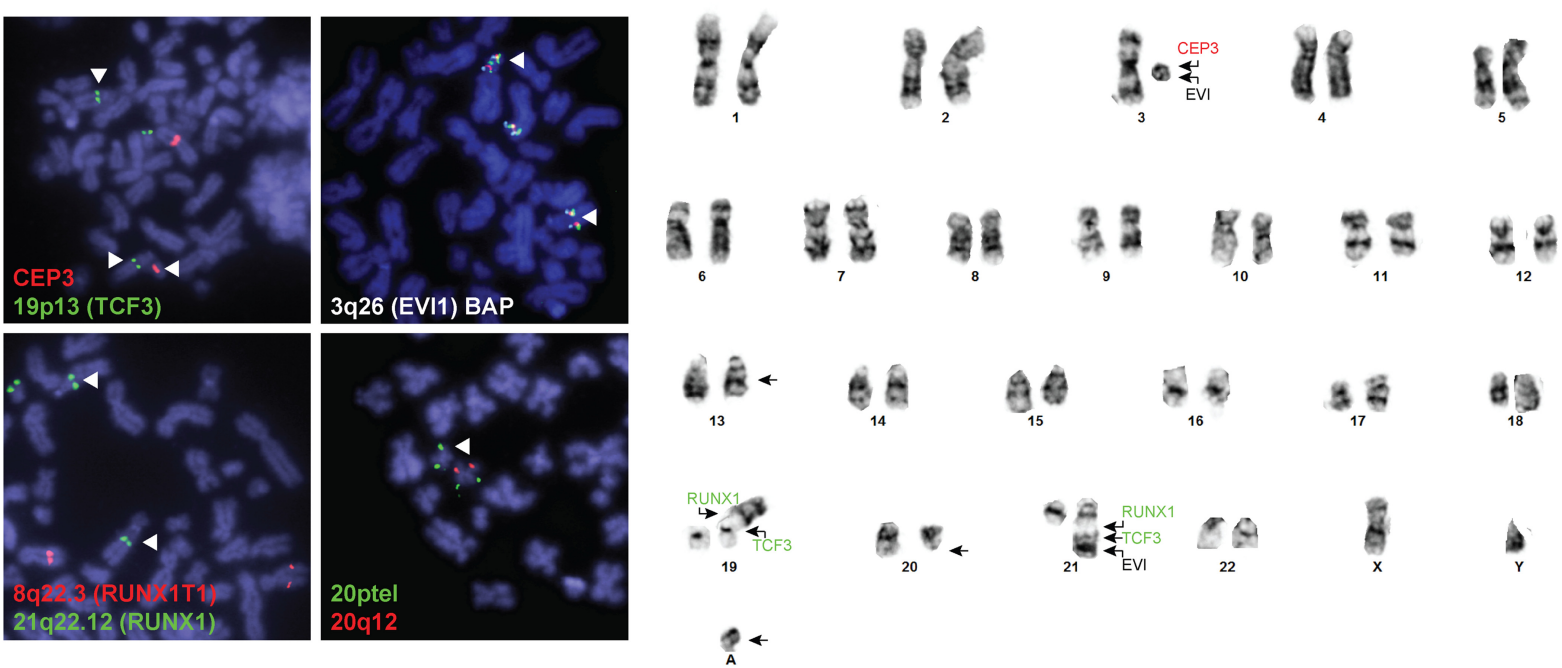
Complex cytogenetics observed in patient 1. The stem line is shown on the right, which was observed in 11 of 21 cells (see Table 3 for complete nomenclature). Fluorescence in situ hybridization (FISH) studies demonstrate the ring chromosome identified by karyotype originates from Chromosome 3 and contains at least the 3q26 locus (*EVI1* gene). The EVI1 probe also localized to a derivative Chromosome 21, which was involved in a three-way translocation with Chromosomes 3 and 19. This was also observed with FISH for RUNX1 (21q22.12), which localized both to the derivative Chromosome 21 and a derivative Chromosome 19. Finally, a terminal deletion of 20q was confirmed in 144/200 interphase cells.

**Figure 3.**
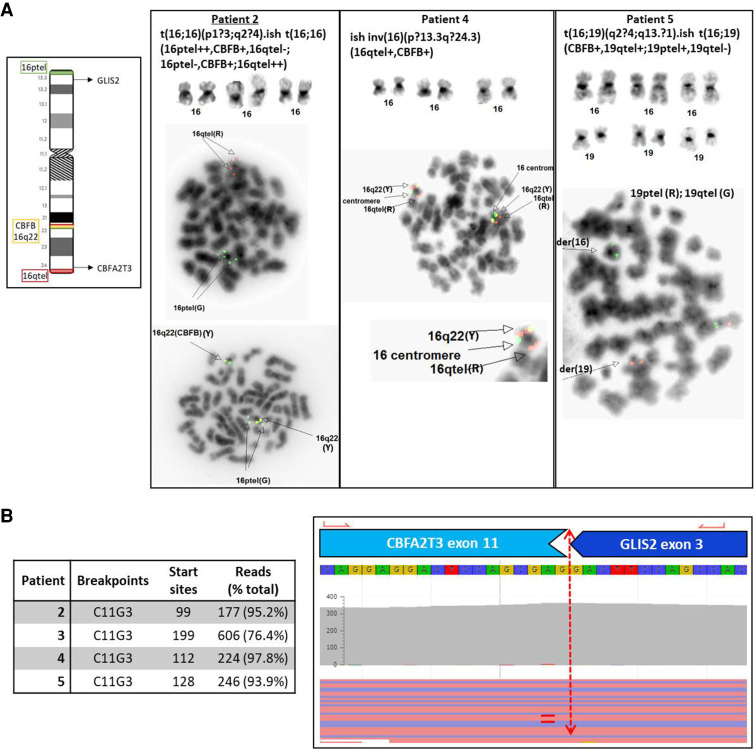
Diverse cytogenetic mechanisms resulting in *CBFA2T3–GLIS2* gene fusions. (*A*) Representative cytogenetic and FISH images used to identify Chromosome 16 abnormalities in patients 2, 4, and 5. See Table 3 for complete nomenclature. Cytogenetic studies are not available for patient 3. (*B*) Representative screenshot from the Archer software demonstrating a *CBFA2T3–GLIS2* gene fusion between exons 11 and 3 of *CBFA2T3* and *GLIS2*, respectively (i.e., C11G3). The same breakpoint was observed in all four patients.

All six patients had comprehensive genomic evaluation (Table 2; Fig. 1), including cytogenetics and comprehensive NGS analyses. Cytogenetic studies revealed multiple changes in all patients, including complex, multiclonal karyotypes in patients 1, 2, and 6 (Table 3). Only patient 4 had a cytogenetic finding highly suggestive of a non-DS AMKL–related translocation, a pericentric inversion associated with *CBFA2T3–GLIS2.* In contrast, in all six patients a gene fusion of diagnostic significance was detected using the NGS panel, including one patient with *NUP98–KDM5A* (Fig. 2), one patient with *KMT2A*–*MLLT6*, and four patients with *CBFA2T3–GLIS2* (Fig. 3). Interestingly, only two patients had clinically significant somatic mutations, both of which were in *SETD2*, a methyltransferase commonly altered in leukemias, typically associated with poor prognosis and which may confer chemoresistance (Table 2; Skucha et al. 2019). Overall, the genomes of these leukemias are characterized by large structural changes resulting in often cryptic, recurrent gene fusions with a relatively low mutational burden (Fig. 1). All five patients with follow-up information available underwent bone marrow transplant because of the high-risk of relapse.

**Table 2.** Genomic characterization

Patient	Specimen	Major cytogenetic findings^a^	Fusions	CNVs by NGS	SNVs^b^ (Tier 1–2), variant allele fraction
1	Bone marrow aspirate	Complex and multiclonal; no pathognomonic findings	*NUP98/KDM5A*	Complex with numerous CNVs, including loss of RB1, and chromothripsis at varying degrees of subclonality^c^	None
2	Bone marrow aspirate	Balanced translocation between Chromosome 16 homologs	*CBFA2T3/GLIS2*	No significant CNV; suboptimal data quality	None
3	Frozen brain mass	Karyotype not performed, FISH inconclusive	*CBFA2T3/GLIS2*	No significant CNV; suboptimal data quality	None
4	Bone marrow aspirate	Pericentric Chromosome 16 inversion	*CBFA2T3/GLIS2*	1q gain	None
5	Bone marrow aspirate	Balanced translocation between long arms of Chromosomes 16 and 19	*CBFA2T3/GLIS2*	No significant CNV; suboptimal data quality	SETD2 p.(Trp1306*), 16%
6	Bone marrow aspirate	Complex and multiclonal with unbalanced translocation between the long arms of Chromosomes 11 and 17	*KMT2A/MLLT6*	No significant CNV	SETD2 p.(Arg441*), 7% EPOR p.(Trp439*) 6%
7	Peripheral blood	Not performed/ordered	NA	Trisomy 21	GATA1 p.(Gln17Valfs*22), 59%
8	Peripheral blood	Constitutional trisomy 21	NA	Trisomy 21	GATA1 p.(Val74Serfs*63), 95%
9	Peripheral blood	NA	NA	Trisomy 21	GATA1 p.(Ser51Leufs*5), 42%
10	Peripheral blood	Constitutional study, PHA-stimulated: trisomy 21	NA	Trisomy 21	GATA1 p.(Glu2Glyfs*38), 73%
11	Bone marrow aspirate	Constitutional trisomy 21 and subclonal trisomies 8 and 14	NA	Trisomy 21	GATA1 p.(Asp65Glyfs*9), 10% RAD21 p.(Tyr3*), 8%
12	Bone marrow aspirate	Constitutional trisomy 21	NA	Trisomy 21	GATA1 p.(Ser30*), 15% CTCF p.(Tyr502Ilefs*10), 7%
13	Bone marrow aspirate	Constitutional trisomy 21 and subclonal trisomy 8	NA	Trisomy 21, subclonal trisomy 8	GATA1 p.(Glu39Glyfs*22), 30% CTCF p.(Asp547Valfs*34), 17% *SUZ12* p.(Leu385Profs*10), 32% JAK2 p.(Phe694Leu), 22%
14	Bone marrow aspirate	Constitutional trisomy 21		Trisomy 21	GATA1 p.(Ser51Leufs*89), 16% GATA1 p.(Ala58Hisfs*14), 38% JAK1 p.(Leu783Phe), 6% *SRSF2* p.(Pro95Arg), 6%
15	Peripheral blood	Constitutional trisomy 21		Trisomy 21	GATA1 p.(Pro73Pro), 16% NRAS p.(Gly13Asp), 17% PTPN11 p.(Thr411Met), 43%

(FISH) Fluorescence in situ hybridization, (NA) not available, (CNV) copy-number variation, (NGS) next-generation sequencing, (SNV) single-nucleotide variation.

^a^See Table 3 for detailed cytogenetic results.

^b^See Table 4 for detailed SNV results.

^c^CNVs characterized by chromosomal microarray instead of NGS.

**Table 3.** Cytogenetic findings

Patient	Specimen	Karyotype	Interphase FISH results
1	Bone marrow aspirate	47,XY,r(3)(::p12−>q12::q24−>q26::),del(13)(q14.1q14.3),der(19)(3pter−>3p12::21q11.2−>21q22::19p13.3−>19qter),del(20)(q11.2q13.1),der(21)(21pter−>21q22::19p13.3−> 19p13.1::3q21−>3qter),+mar[11].ish r(3)(CEP3+,EVI1+), der(19)(RUNX1+,TCF3+), del(20)(D20S108−), der(21)(RUNX1+,TCF3+,EVI1+)/46,sl,t(14;18) (p11.1;q11.2), −mar[5]/46,sl, der(13)(?21pter−>21q21::13q11.2−>13q14.1::13q14.3 −> 13q34::13q14.3−>13qter), −del(13), −mar[5]	nuc ish(EVI1 × 3)[150/200] nuc ish(RUNX1T1 × 2,RUNX1 × 3)[146/200] nuc ish(TCF3 × 3,CEP3 × 2)[150/200] nuc ish(20ptelx2,D20S108 × 1)[144/200]
2	Bone marrow aspirate	46,XX,t(16;16)(p1?3;q2?4),der(22)t(1;22)(q10;q10)[6].ish t(16;16)(16ptel++,CBFB+,16qtel−;16ptel−,CBFB+, 16qtel++)/46,idem,i(17)(q10)[5]/46,XX[8]	nuc ish(RUNX1T1,RUNx1)x2[200] nuc ish(CBFB,16ptel)x2[200]
3	Frozen brain mass	Not performed/ordered	nuc ish(EVI1 × 2)[199/200] nuc ish(PDGFRB)x3[9/200] nuc ish(CEP7,D7S486)x2[200] nuc ish(KMT2Ax4)(5′KMT2A sep 3′KMT2Ax1)[5/200] nuc ish(PML,RARA)x2[200] nuc ish(CBFBx2)[200]
4	Bone marrow aspirate	46,XY,der(14)t(1;14)(q2?1;q32)[13].ish der(14)(LSI1q25+,IGH−),inv(16)(G248P8680H10+,CBFB+)/46,XY[7]	nuc ish(LSI1p36 × 2,LSI1q25 × 3)[110/200] nuc ish(CEP8 × 2,MYCx2,IGHx1)[116/200] nuc ish(KMT2Ax2)[200]
5	Bone marrow aspirate	47,XX,del(7)(q2?2),?t(16;19)(q2?4;q13.?1),+21[7].ish del(7)(CEP7+,D7S486−),t(16;19) (CBFB+,G248P85334F2+;G248P89955D12+,G248P85334F2−)/46,XX[13]	nuc ish(CEP7 × 2,D7S486 × 1)[54/200] nuc ish(RUNX1T1 × 2,RUNX1 × 3)[50/200] nuc ish (KMT2Ax2)[200] nuc ish(CBFBx2)[200]
6	Bone marrow aspirate	46,XY,t(11;17)(q23;q2?1)[1]/49,idem,+2,+5,+21[6]/47,XY,der(5)t(5;11)(q12;p15),der (11)t(5;11)(q12;p15)t(11;17)(q23;q2?1),der(17)t(11;17)(q23;q2?1),+19[10]/46,XY[4].ish t(11;17)(5′KMT2A+,3′KMT2A−;5′KMT2A+,3′KMT2A+)	nuc ish(5′KMT2Ax3,3′KMT2Ax2)(5′KMT2A con 3′KMT2Ax2)[50/200], (ETV6 × 2,RUNX1 × 3)[38/200]
7	Peripheral blood	Not performed / ordered	NA
8	Peripheral blood	47,XY,+21c[19]	nuc ish(CBFBx2)[200]
9	Peripheral blood	NA	NA
10	Peripheral blood	47,XY,+21 (constitutional study, PHA-stimulated)	NA
11	Bone marrow aspirate	49,XY,+8,+14,+21c[10]/46,XY,+21c[10]	nuc ish(CEP8,MYC,IGH)x3[50/200]
12	Bone marrow aspirate	47,XY,?del(3)(q21q25),del(7)(p13),+21c[16].ish ?del(3)(EVI1+,BCL6+)/47,XY,+21c[14]	nuc ish(EVI1 × 2)[200] nuc ish(BCL6 ×× 2)[200] nuc ish(CEP7,D7S486)x2[200]
13	Bone marrow aspirate	48,XY,+8,+21c[14]/47,XY,+21c[7]	nuc ish(RUNX1T1 × 3,RUNX1 × 3)[125/200]/(RUNX1T1 × 2,RUNX1 × 3)[75/200]
14	Bone marrow aspirate	47,XY,+21c[20]	nuc ish(PDGFRB)x2[200] nuc ish(CEP7,D7S486)x2[200] nuc ish(RUNX1T1 × 2,RUNX1 × 3)[195/200] nuc ish(MLLx2)[200] nuc ish(PML,RARA)x2[200] nuc ish(CBFBx2)[200]
15	Peripheral blood	47,XX,+21c[20]	nuc ish(PDGFRB)x2[200] nuc ish(CEP7,D7S486)x2[200] nuc ish(RUNX1T1 × 2,RUNX1 × 3)[200] nuc ish(MLLx2)[200] nuc ish(CBFBx2)[200]

(FISH) Fluorescence in situ hybridization, (NA) not applicable.

### Down Syndrome–Related Transient Abnormal Myelopoiesis (TAM)

**Figure 4.**
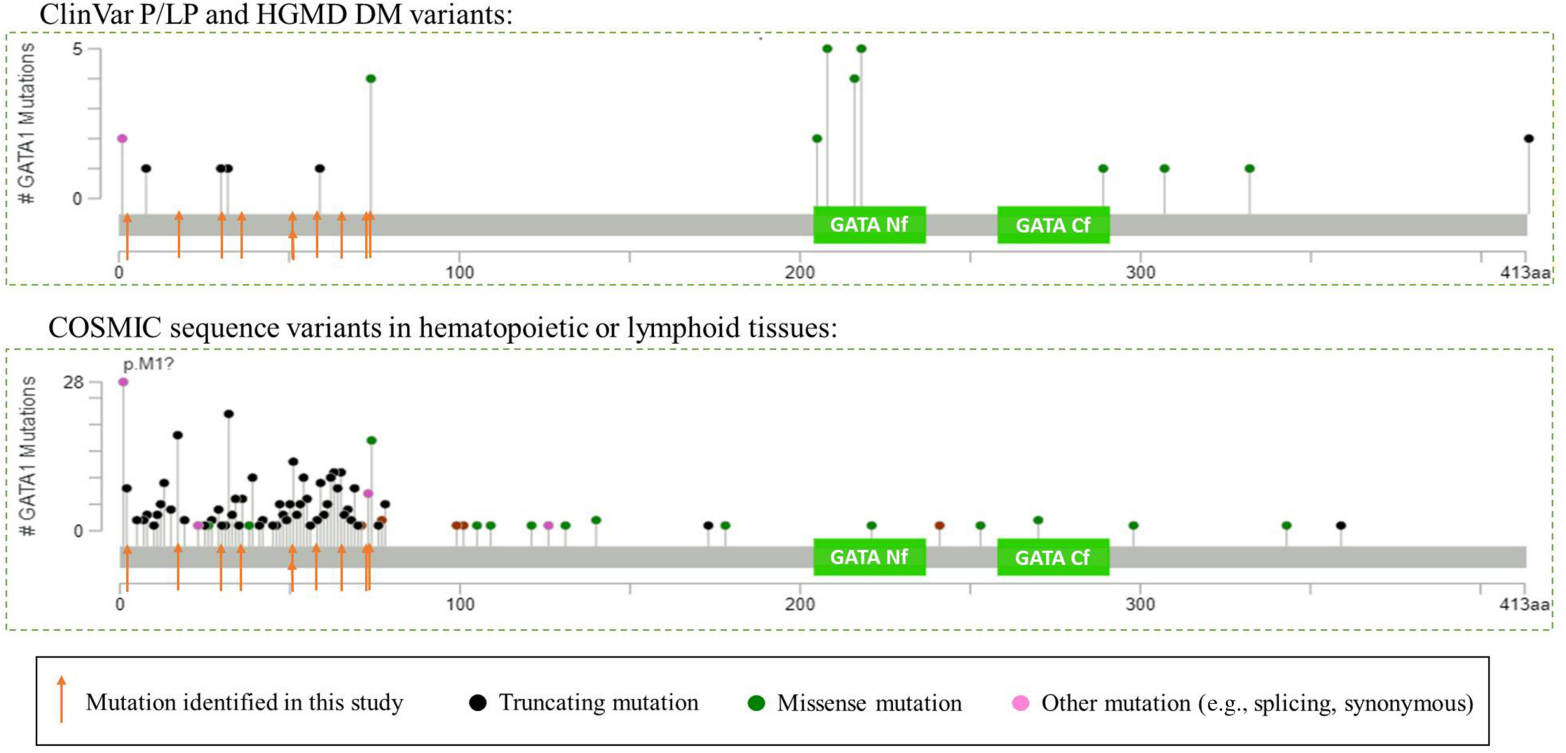
*GATA1* variant spectrum in germline and somatic setting. The green boxes indicate the two GATA zinc finger protein domains, the amino-terminal finger (Nf) and carboxy-terminal finger (Cf). (*A*) *GATA1* variants listed in ClinVar as Pathogenic or Likely Pathogenic or in HGMD as “disease-associated” for hereditary conditions. (*B*) *GATA1* sequence variants listed in COSMIC identified in hematopoietic or lymphoid tissues. Intronic variants that may affect splicing are not included. Numbering based on RefSeq transcript NM_002049.3.

Four patients in our cohort were diagnosed with TAM and presented between 2 d to 6 wk of life (Table 1). As expected, a *GATA1* SNV was observed in each patient, and no fusion events were observed (Table 2; Fig. 4). The *GATA1* mutations were present at high variant allele fraction (VAF), ranging from 42% to 95%.

Despite similar genetic profiles, significantly different clinical outcomes were observed (clinical details not available for patient 7). Patients 8 and 9 presented with high-risk features including leukocytosis and respiratory distress and were thus treated with low-dose cytarabine according to standard protocols (Gamis et al. 2011). Patient 8 developed significant comorbidities including ascites, hepatic dysfunction, and disseminated intravascular coagulation and ultimately passed away at 3 mo of age because of multiorgan failure secondary to his complete atrioventricular canal. Patients 9 and 10 remain leukemia-free at 20 mo and 1 yr of age, respectively.

### Down Syndrome–Related AMKL (DS-AMKL)

In contrast to the patients with TAM, the five patients diagnosed with DS-AMKL presented as toddlers (14–25 mo), and only patient 12 had a known prior history of TAM. Patients were treated according to the Children's Oncology Group (COG) AAML1531 protocol, and all patients remain disease-free (follow-up time 8.5–30 mo).

The genetic profiles in these DS-AMKL patients were distinct from patients with TAM or non-DS AMKL (Fig. 1). Although all patients had *GATA1* mutations, they also had additional molecular and/or cytogenetic changes (range two to four additional changes; Table 2). Two patients had trisomy 8, patient 12 had other cytogenetic changes, and all five patients had sequence mutations in pathways known to be involved in DS-AMKL, including two with frameshift mutations in *CTCF*. The other mutations involve genes in the RAS pathway (*JAK1, JAK2, NRAS, PTPN11*), cohesin complex (*RAD21),* epigenetic regulators (*SUZ12*), and spliceosome components (*SRSF2)*.

## DISCUSSION

In our cohort, the three groups of patients displayed distinct genomic profiles (Fig. 1). Non-DS AMKL is characterized by large cytogenetic changes, often with complex genomes, recurrent gene fusions, and a paucity of sequencing mutations. TAM and DS-AMKL are characterized by trisomy 21 and *GATA1* frameshift mutations with additional cytogenetic and sequence mutations observed in DS-AMKL exclusively.

### Genomic-Guided Management in Non-DS AMKL

Although a diagnosis of non-DS AMKL has been historically associated with poor prognosis, these patients are clinically heterogeneous, and recent studies identified recurrent genomic changes that delineate patients into distinct prognostic groups. Inaba et al. (2015) defined three risk groups based on karyotypes, with high-risk patients having normal karyotypes, −7, 9p abnormalities, including t(9;11)(p22;q23), −13/13q−, or −15. Low-risk patients have 7p abnormalities whereas the remaining patients are considered intermediate risk. More recently, recurrent gene fusions have been used to define prognosis with *NUP98–KDM5A*, *CBFA2T3–GLIS2*, and *KMT2A* rearrangements being associated with poor prognosis, *HOX* rearrangements and *RBM15–MKL1* being associated with intermediate prognosis, and *GATA1* mutations being associated with good prognosis (de Rooij et al. 2016, 2017). Current risk stratification in COG is based on specific cytogenetic/molecular features, with allogenic hematopoietic stem cell transplantation (HSCT) offered during the first remission for patients with high-risk AML (Elgarten and Aplenc 2020).

The non-DS AMKL-associated gene fusions have been shown to be important drivers of leukemogenesis associated with distinct transcriptional programs and clinical outcomes (de Rooij et al. 2017). For instance, both *CBFA2T3* and *GLIS2* are transcriptional regulators, and the *CBFA2T3–GLIS2* fusion results in increased Hedgehog, JAK-STAT, and growth factor signaling pathways leading to enhanced self-renewal of hematopoietic stem cells (Gruber et al. 2012; Thiollier et al. 2012). Interestingly, we identified this fusion in four of six patients with non-DS AMKL, indicating it may be more common than previously thought in pediatric patients, although our study is limited in size (see Study Limitations below). Our panel may have a higher detection rate for this fusion because of the open-ended polymerase chain reaction (PCR) technology used and increased sensitivity of using a targeted fusion panel as opposed to whole-transcriptome sequencing, which may be less efficient for fusions with lower expression.

In some cases, a diagnosis of AMKL cannot be achieved based on morphological or immunophenotypic information alone, and comprehensive genomic evaluation can reveal recurrent AMKL-associated mutations allowing for an integrated diagnosis of non-DS AMKL. Patients 1 and 3 did not receive a definitive diagnosis until genetic testing revealed *NUP98–KDM5A* and *CBFA2T3–GLI3* gene fusions, respectively. Patient 1 was initially diagnosed with undifferentiated AML, and upon identification of the fusion gene, his diagnosis was changed to non-DS AMKL. Additionally, the complex cytogenetic aberrations observed further confirmed a poor prognosis for this patient. For patient 3, although flow cytometry revealed megakaryocytic differentiation, he presented with a temporal sarcoma, which complicated diagnosis. Evaluation of bone marrow and correlation with genetic features confirmed a diagnosis of non-DS AMKL. Because children with AML and *NUP98* or *CBFA2T3* fusions treated with chemotherapy alone have poor relapse-free survival (de Rooij et al. 2016), both patients underwent a matched unrelated donor bone marrow transplant.

### Significance of Genomic Evaluation in Patients with DS

Even within DS-related myeloid malignancy, the clinical heterogeneity is striking. Despite the fact that all patients with TAM acquire *GATA1* mutations, it has been estimated that up to two-thirds remain asymptomatic (i.e., silent TAM), whereas the remaining one-third (∼10% of newborns with DS) develop clinical TAM (Roberts et al. 2013; Swerdlow et al. 2017). In most patients, TAM resolves spontaneously; however, in 20%–30% of patients, TAM transforms to myelodysplastic syndrome (MDS) or AML, most commonly DS-AMKL, 1–3 yr later. One theory postulates that the initiating *GATA1* clone occurs in tissues of fetal hematopoiesis, which could explain the spontaneous remission observed in most patients with TAM within the first year of life, when bone marrow hematopoiesis takes over (Bhatnagar et al. 2016). If additional driver mutations arise in these clones prior to extinction of fetal hematopoiesis stem cells, there is risk of DS-AMKL. In our cohort, only one of the five patients with DS-AMKL was known to have TAM prior to their AMKL diagnosis, suggesting the remaining four had silent TAM and accumulated additional driver mutations prior to extinction of fetal hematopoiesis stem cells. Given that *GATA1* is located on the X chromosome, it is interesting to note that eight of the nine patients with TAM or DS-AMKL in this cohort are male, consistent with previous studies that noted a trend toward sex bias in development of TAM/DS-AMKL (Massey et al. 2006; Heald et al. 2007). Whether the variant allele frequency of *GATA1* mutations is associated with development and/or progression to DS-AMKL requires additional studies.

The acquired *GATA1* mutations in TAM/DS-AMKL are predominantly frameshift mutations in exon 2 (NM_002049.3), resulting in expression of a shorter isoform due to an alternative translation initiation codon at residue 84, thereby skipping the frameshift mutations and the boundary between exon 2 and intron 2. These mutations may be embryonic lethal as they have rarely been reported as germline changes (Rainis et al. 2003). In contrast, pathogenic germline variants in *GATA1* are mostly missense mutations in exon 4 (NM_002049.3) encoding the amino-terminal zinc finger protein domain (also known as the Nf domain, residues 204–237 based on UniProtKB reference P15976) and are associated with X-linked anemia, neutropenia, thrombocytopenia, and platelet abnormalities, but not leukemias (Fig. 4) (Ciovacco et al. 2008).

All DS-AMKL harbored additional somatic mutations, consistent with a prior exome sequencing study showing a higher mutation burden in individuals with DS-AMKL compared to TAM (Yoshida et al. 2013). All patients with DS-AMKL had at least one additional driver mutation with an enrichment in cohesion genes, epigenetic regulators, *CTCF*, and other critical signaling pathway genes (Nikolaev et al. 2013; Bhatnagar et al. 2016). Here, we identify the second DS-AMKL patients with somatic mutations in *SUZ12* and *SRSF2*, respectively (Yoshida et al. 2013). Of note, two *GATA1* mutations were observed in patient 14 (DS-AMKL), which likely arose independently in distinct clonal populations; indeed visualization in the Integrated Genomics Viewer confirmed that these two variants were mutually exclusive. This finding is consistent with the theory that cells with trisomy 21 have a “mutator phenotype” specific to certain loci such as *GATA1* (Roberts et al. 2013). In two published individuals with more than one *GATA1* mutation and genetic profiling at the time of TAM and DS-AMKL diagnoses, the minor TAM subclone expanded and acquired additional mutations to become the major DS-AMKL clone (Nikolaev et al. 2013; Yoshida et al. 2013).

Identifying patients with TAM at greatest risk of DS-AMKL is not evident a priori as these patients have indistinguishable pathology at the time of initial diagnosis. The natural history study by the COG evaluated multiple clinical risk factors and only time to TAM resolution reached borderline significance with leukemia-free survival (Gamis et al. 2011). Of note, neither *GATA1* mutation type nor diagnostic blast count is associated with progression to AMKL (Gamis et al. 2011; Bhatnagar et al. 2016). An outstanding question is whether serial, high-depth sequencing of genes involved in TAM to AMKL progression may help in early detection of DS-AMKL transformation.

### Importance of Comprehensive Evaluation

Accurate and timely diagnosis and prognostication require multiple molecular and cytogenetic techniques, combining fast turnaround times with thorough evaluation. At the moment, clinical laboratories utilize various techniques to offer such comprehensive testing; however, RNA studies, which are required to detect most AMKL-related gene fusions, are not considered standard of care.

Detection of *GATA1* mutations is important for diagnosis of TAM, especially “silent” TAM in which infants have a small fraction of clones with *GATA1* mutations but no clinical phenotype (Roberts et al. 2013). These infants are at increased risk of developing DS-AMKL. Pending studies will determine whether periodic mutation screening can improve outcomes for these infants with “silent” TAM and thus the clinical utility of screening for *GATA1* mutations in all patients with DS (Roberts et al. 2013; Bhatnagar et al. 2016).

Cytogenetic evaluation can rapidly identify canonical chromosomal rearrangements associated with non-DS AMKL as well as identify prognostic structural changes. For example, a pericentric inversion of Chromosome 16 was detected with cytogenetics in the bone marrow of patient 4, strongly suggesting a *CBFA2T3–GLIS2* gene fusion, which was subsequently confirmed with the NGS RNA panel. However, many of the recurrent rearrangements associated with AMKL are cytogenetically cryptic and were detected using the RNA sequencing only. Indeed, despite four patients harboring the recurrent *CBFA2T3–GLIS2* with standard breakpoints, only one displayed the typical cytogenetic mechanism, a pericentric inversion 16 (patient 4; Fig. 3). In the remaining two patients with this fusion with karyotype analysis, one was caused by a balanced translocation between the two Chromosome 16 homologs and the other likely involved a complex rearrangement in which only a balanced translocation between Chromosomes 16 and 19 could be observed (patient 5).

### Study Limitations

One caveat to this study is the possibility of missing fusion genes involving genes of the *HOX* gene cluster, which are not included in the current RNA panel. However, some partner genes that have been observed in *HOX* fusions are included, such as *EWSR1*. Based on prior studies using unbiased transcriptomic profiling, it is expected that ∼15% of patients with non-DS AMKL carry *HOX* rearrangements, which are associated with intermediate prognosis. Alternatively, our cohort may be biased toward patients with aggressive disease referred to the Children's Hospital of Philadelphia. This is supported by the lack of patients with genetic features associated with good (*GATA1* somatic mutations) or intermediate (*HOX*-rearrangements and *RBM15–MKL1* fusion) prognosis. Indeed, an electronic medical record search for pathological features of AMKL did not reveal any additional patients.

In summary, AMKL-related malignancies display both clinical and genomic heterogeneity. Recurrent molecular and cytogenetic changes can predict prognosis and guide management decisions. High-throughput DNA and RNA profiling is required for optimal management of AMKL-related malignancies, whereas cytogenetic studies can provide diagnostic results in a short time frame and may help to reveal underlying mechanism of genomic alterations. Thus, integrated genomic diagnostic enables personalized patient care.

## METHODS

### Patient Characteristics

Patients were identified by pathological diagnosis and/or molecular findings. Comprehensive genomic evaluation using a large NGS panel for DNA and RNA has been offered at the Children's Hospital of Philadelphia since 2016. This clinical cohort was mined for patients with a pathological or molecular diagnosis of AMKL, TAM, or DS-AMKL. Specifically, the pathology reports of patients having undergone genomic profiling were mined for the words “AMKL,” “megakaryoblastic,” “CD41,” or “CD61.” Additionally, an internal genomic database was mined for clinical indications related to AMKL, TAM, or DS-AMKL and for genetic findings associated with these malignancies, including *GATA1* mutations and the recurrent gene fusions observed in AMKL. This study was approved by the Institutional Review Board of the Children's Hospital of Philadelphia.

### Cytogenetics

Chromosome analysis and FISH studies were performed according to standard protocols. Briefly, unstimulated bone marrow specimens were cultured and harvested after overnight incubation. G-banding metaphases were prepared using trypsin digestion followed by Giesma staining. A minimum of 20 cells were analyzed. FISH studies were performed using a panel of probes for AML and additional probes may be added based on clinical indication, where appropriate, to rule out recurrent abnormalities.

### NGS Panel

The Comprehensive Hematological Cancer Panel offered by the Children's Hospital of Philadelphia Division of Genome Diagnostics (CAP-accredited) is a custom panel including sequence and copy-number analyses of 99 (version 1) to 118 (version 2) cancer genes and gene fusion detection involving one of 106 (version 1) to 110 (version 2) possible partner genes (exact number depends on date of service). The genes included in the current panel are listed at https://apps.chop.edu/service/laboratories/olsd.cfm/division-genomic-diagnostics. DNA and RNA extraction, sequencing, and analysis were performed as previously reported (Chang et al. 2019; Surrey et al. 2019). Briefly, DNA sequencing libraries are prepared from 50 ng genomic DNA using the Agilent SureSelect^QXT^ kit, whereas RNA libraries are prepared from 150 ng input RNA or total nucleic acid using the Archer Universal RNA Reagent Kit v2. All sequencing reactions are performed with the Illumina MiSeq or HiSeq platforms with paired-end sequencing (2 × 150 bp). Average sequencing coverage for the region of interest of the DNA panel is 1500× and minimum sequencing coverage is 100×. See Supplemental Table 1 for sequencing and alignments metrics. In-house scripts are used to identify and annotate single nucleotide variants (SNVs) and small insertion-deletions (indels) detected within exonic regions ±10 bp flanking regions and intonic regions with known mutations, and copy-number variants (CNVs) are analyzed using NextGENe v2 NGS Analysis Software (Softgenetics) (Surrey et al. 2019). Gene fusions are detected using Archer Analysis according to standard protocols (Chang et al. 2019).

Somatic mutations are classified using criteria consistent with those recommended by the Association for Molecular Pathology, American Society of Clinical Oncology, and College of American Pathologists (Li et al. 2017). In brief, Tier 1 variants are actionable somatic variants with well-established evidence for diagnostic, prognostic or therapeutic implications. Tier 2 variants represent potentially actionable somatic variants. Tier 3 and 4 variants represent variants of unknown significance and likely benign/benign variants, respectively. Only Tier 1–2 variants are reported in this manuscript. Variants in *GATA1* are reported based on transcript NM_002049.3, and transcripts for remaining variants described are listed in Table 4.

**Table 4.** Detailed sequencing variants results

Patient	Gene	Chromosome	HGVS DNA	HGVS protein	Variant type	COSMIC ID	VAF	Variant classification
5	*SETD2* (NM_014159.6)	Chr 3:47162208	c.3918G > A	p.(Trp1306*)	Nonsense	COSM5574965	16%	Tier 2
6	*SETD2* (NM_014159.6)	Chr 3:47164805	c.1321C > T	p.(Arg441*)	Nonsense	COSM3736817	7%	Tier 2
6	*EPOR* (NM_000121.3)	Chr 19:11488871	c.1316G > A	p.(Trp439*)	Nonsense	-	6%	Tier 2
7	*GATA1* (NM_002049.3)	Chr X:48649565	c.49_50del	p.(Gln17Valfs*22)	Frameshift	COSM13216	59%	Tier 1
8	*GATA1* (NM_002049.3)	Chr X:48649735	c.219delA	p.(Val74Serfs*63)	Frameshift	-	95%	Tier 1
9	*GATA1* (NM_002049.3)	Chr X:48649666	c.151_186delinsT	p.(Ser51Leufs*5)	Frameshift	COSM17631	42%	Tier 1
10	*GATA1* (NM_002049.3)	Chr X:48649520	c.4dup	p.(Glu2Glyfs*38)	Frameshift	-	73%	Tier 1
11	*GATA1* (NM_002049.3)	Chr X:48649689	c.175_193dup	p.(Asp65Glyfs*9)	Frameshift	COSM6907368	10%	Tier 1
11	*RAD21* (NM_006265.2)	Chr 8:117878960	c.9C > A	p.(Tyr3*)	Nonsense	COSM3663527	8%	Tier 2
12	*GATA1* (NM_002049.3)	Chr X:48649605	c.89C > G	p.(Ser30*)	Nonsense	COSM17632	15%	Tier 1
12	*CTCF* (NM_006565.3)	Chr 16:67660601	c.1502_1503dupAT	p.(Tyr502Ilefs*10)	Frameshift	-	7%	Tier 2
13	*GATA1* (NM_002049.3)	Chr X:48649633	c.115_116ins64	p.(Glu39Glyfs*22)	Frameshift	-	30%	Tier 1
13	*CTCF* (NM_006565.3)	Chr 16: 67662394	c.1640_1650delinsT	p.(Asp547Valfs*34)	Frameshift	-	17%	Tier 2
13	*SUZ12* (NM_015355.3)	Chr 17:30315465	c.1150_1151delAG	p.(Leu385Profs*10)	Frameshift	-	32%	Tier 2
14	*GATA1* (NM_002049.3)	Chr X:48649658	c.142_149dup	p.(Ser51Leufs*89)	Frameshift	-	16%	Tier 1
14	*GATA1* (NM_002049.3)	Chr X:48649675	c.159_171dup	p.(Ala58Hisfs*14)	Frameshift	-	38%	Tier 1
14	*JAK1* (NM_002227.3)	Chr 1:65309803	c.2347C > T	p.(Leu783Phe)	Missense	COSM41758	6%	Tier 2
14	*SRSF2* (NM_003016.4)	Chr 17:74732959	c.284C > G	p.(Pro95Arg)	Missense	COSM4385016	6%	Tier 2
15	*GATA1* (NM_002049.3)	Chr X:48649735	c.219A > G	p.(Pro73Pro)	Splicing (predicted)	COSM17826	16%	Tier 1
15	*NRAS* (NM_002524.4)	Chr 1:115258744	c.38G > A	p.(Gly13Asp)	Missense	COSM573	17%	Tier 2
15	*PTPN11* (NM_002834.3)	Chr 12:112924286	c.1232C > T	p.(Thr411Met)	Missense	COSM4038882	43%	Tier 2

Chromosome coordinates are based on the hg19 reference.

(HGVS) Human Genome Variation Society, (COSMIC) Catalogue of Somatic Mutations in Cancer, (VAF) variant allele frequency.

## ADDITIONAL INFORMATION

### Data Deposition and Access

All clinically relevant variants (classified as Tier 1–Tier 3) are included in this article and its Supplemental Files. These variants were deposited to ClinVar (https://www.ncbi.nlm.nih.gov/clinvar/) and can be found under variant accession numbers RCV001293744–RCV001293768. Raw data sets are not available on public databases because of lack of patient consent for data deposition (see below). Additional information is available from the corresponding author, when appropriate.

### Ethics Statement

This study was approved by the Children's Hospital of Philadelphia Institutional Review Board as a retrospective analysis (IRB 17-013802). The IRB granted a waiver of consent and assent because of the retrospective nature of this study and no more than minimal risk (breach of confidentiality) to subjects. However, this waiver of consent or assent does not allow depositing full/raw data sets into publicly available databases because of risk of breach of confidentiality. This study was conducted in accordance with the Declaration of Helsinki.

### Acknowledgments

The authors thank all members of the Division of Genome Diagnostics at the Children's Hospital of Philadelphia.

### Author Contributions

All authors have significantly contributed to this manuscript and have read approved this submission to *Cold Spring Harbor Molecular Case Studies*. E.L. collected and analyzed the data and wrote the manuscript. S.R. analyzed genetic data and contributed to figures. G.W., L.F.S., F.L., X.Z., M.L., and M.M.L. analyzed genetic data. A.O. and R.A. collected data. E.L., M.L., and M.M.L. designed the study.

### Funding

This work was supported by the Children's Hospital of Philadelphia.

### Competing Interest Statement

M.M.L. is on the SAB of Roche Sequencing Solutions. The rest of the authors disclose no conflicts of interest.

## Supplementary Material

Supplemental Material
