# Supplementary material for: Clinical impact of genomic characterization of 15 patients with acute megakaryoblastic leukemia–related malignancies
Source: Cold Spring Harb Mol Case Stud. 2021 Apr;7(2):a005975. doi: 10.1101/mcs.a005975 (PMC8040732; doi:10.1101/mcs.a005975)
Supplement: Supplemental Material [file supp_7_2_a005975__index.html]

Supplemental Material 

# Clinical impact of genomic characterization of 15 patients with acute megakaryoblastic leukemia–related malignancies

## Supplemental Material

- Supplemental\_Table\_1.docx
