# Supplementary material for: Clinical impact of genomic characterization of 15 patients with acute megakaryoblastic leukemia–related malignancies
Source: Cold Spring Harb Mol Case Stud. 2021 Apr;7(2):a005975. doi: 10.1101/mcs.a005975 (PMC8040732; doi:10.1101/mcs.a005975)
Supplement: Supplemental Material [file supp_mcs.a005975_Supplemental_Table_1.docx]

Supplementary Table 1. Sequencing and alignment metrics for the targeted DNA panel.

| **Patient** | **Sequencing_ Platform** | **Total Reads** | **% Reads  Aligned** | **Average ROI  Coverage** | **% ROI 1x** | **% ROI 80x** | **% ROI 100x** |
| --- | --- | --- | --- | --- | --- | --- | --- |
| 1 | HiSeq | 21793954 | 98.42 | 2604.55 | 100 | 99.3 | 98.9 |
| 2 | HiSeq | 26014846 | 99.32 | 2809.12 | 100 | 97.6 | 96.8 |
| 3 | HiSeq | 25544034 | 98.87 | 3174.37 | 100 | 99.7 | 99.6 |
| 4 | HiSeq | 22276376 | 97.94 | 2817.33 | 100 | 99.4 | 99.2 |
| 5 | HiSeq | 26023926 | 99.03 | 3342.5 | 100 | 99.6 | 99.5 |
| 6 | HiSeq | 21983314 | 99.24 | 2345.29 | 100 | 99.4 | 99.1 |
| 7 | HiSeq | 22434964 | 99.05 | 2442.44 | 100 | 100 | 100 |
| 8 | HiSeq | 23607122 | 98.57 | 2532.49 | 100 | 98.2 | 97.5 |
| 9 | HiSeq | 26882660 | 99.27 | 3245.75 | 100 | 100 | 100 |
| 10 | HiSeq | 27074764 | 99.20 | 2931.67 | 100 | 99.5 | 99.1 |
| 11 | HiSeq | 25579350 | 99.20 | 3085.5 | 100 | 99.6 | 99.2 |
| 12 | HiSeq | 27007808 | 99.30 | 3171.6 | 100 | 99.7 | 99.5 |
| 13 | MiSeq | 11005176 | 95.91 | 1484.22 | 100 | 99.9 | 99.8 |
| 14 | MiSeq | 12951472 | 97.87 | 1999.45 | 100 | 100 | 100 |
| 15 | MiSeq | 7967448 | 99.25 | 1300.63 | 100 | 99.9 | 99.9 |

ROI: region of interest
